# Supplementary material for: Cigarette Smoke Condensate Exposure Induces Receptor for Advanced Glycation End-Products (RAGE)-Dependent Sterile Inflammation in Amniotic Epithelial Cells
Source: Int J Mol Sci. 2021 Aug 3;22(15):8345. doi: 10.3390/ijms22158345 (PMC8348034; doi:10.3390/ijms22158345)
Supplement: Supplementary file 1 [file ijms-22-08345-s001.zip › ijms-1303108-supplementary.pdf]

Amniotic epithelial cells (48h)

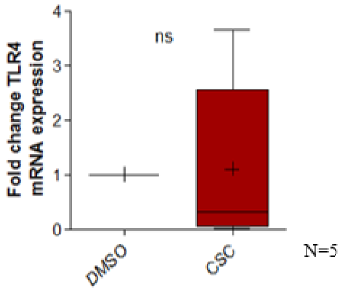

Supplementary data S1:
CSC impact on TLR4 expression in amniotic epithelial cells
(Choltus *et al.* 2021)

TLR4 transcripts expression was measured in amniotic epithelial cells treated or not with CSC during 48h (N=5). Statistical analysis was performed thanks to a non-parametric t-test Mann-Whitney. ns means not significative.
